# Sunitinib in Metastatic Renal Cell Carcinoma: The Pharmacological Basis of the Alternative 2/1 Schedule

**DOI:** 10.3389/fphar.2017.00523

**Published:** 2017-08-07

**Authors:** Antonello Di Paolo, Sergio Bracarda, Elena Arrigoni, Romano Danesi

**Affiliations:** ^1^Department of Clinical and Experimental Medicine, University of Pisa Pisa, Italy; ^2^Medical Oncology, Ospedale San Donato USL8, Istituto Toscano Tumori Arezzo, Italy

**Keywords:** sunitinib, metastatic renal cell carcinoma, efficacy, tolerability, pharmacokinetics, pharmacodynamics

## Introduction

Sunitinib is an inhibitor of platelet-derived growth factors receptor (PDGFR) and vascular endothelial growth factor receptor-2 (VEGFR2) tyrosine-kinase activities, and it is registered for the treatment of metastatic renal cell carcinoma (mRCC). Indeed, sunitinib 50 mg/day for 28 days every 6 weeks is effective against mRCC and induces significant improvements in progression-free and overall survival (PFS and OS, respectively) with respect to interferon-α and interleukin-2, even in cytokine-resistant tumors (Motzer et al., 2006a,b; Motzer et al., 2007). Moreover, in some patients (30–50%) sunitinib efficacy is counterbalanced by toxicities (Najjar et al., 2014), which are mainly represented by thrombocytopenia (10%), fatigue (9%), asthenia, neutropenia, and hand-foot syndrome (each 7%) (Gore et al., 2015). Toxic effects usually worse between weeks 3 and 4 of therapy, requiring treatment interruption in approximately one-third of patients (van der Veldt et al., 2008; Najjar et al., 2014). The most severe toxicities are associated with low body surface area, older age, and female gender (van der Veldt et al., 2008). Intriguingly, those three factors could be potentially related to increased sunitinib exposure, because the drug is administered as a fixed dose without any adjustment. Indeed, the apparent drug clearance was slightly reduced (approximately 8%) in women with respect to men, while the body weight influenced the apparent volume of distribution (Houk et al., 2009). Those results could be the basis for patients' stratification, but the individualization of the dose appeared to be difficult because those variables were clinically evident only in combination (i.e., a thin woman) and the inter-patient variability in pharmacokinetic parameters was about 40–60% (Houk et al., 2009). The third obstacle to dose individualization is represented by the non-linear pharmacokinetics of sunitinib, “making dose-response modeling challenging” (Houk et al., 2010).

Therefore, the occurrence of moderate-to-severe toxicities seems related to changes/alterations in drug pharmacokinetics in particular subgroups of patients, but dose individualization seems still difficult. In the very recent past, several studies have described a modified schedule for the administration of sunitinib, 50 mg/day for 14 days every 3 weeks (2/1 schedule) instead of the standard 4/2 schedule (4 weeks on treatment and 2 weeks of rest). The new schedule has the same dose intensity with respect the classical one, but tolerability has significantly increased (Bracarda et al., 2015). Those evidence and hypothesis find solid pharmacological bases.

## Pharmacokinetics and pharmacodynamics of sunitinib in mRCC

The modulation of molecular targets (i.e., the inhibition of VEGFR2 and PDGFR) in preclinical model was dependent on the dose (Mendel et al., 2003). Doses lower than 80 mg/kg were unable to completely inhibit receptor activation, and that effect disappeared within 8–12 h from the exposure. However, sunitinib did not require the constant inhibition of both receptors to exert a potent antitumor effect (Mendel et al., 2003). Indeed, the vascular permeability was highly inhibited hours after the cessation of sunitinib treatment while the standard dose of 50 mg/day resulted in plasma concentrations within the target interval of 50–100 ng/mL (Faivre et al., 2006) that were associated with therapeutic benefits (Gore et al., 2015).

In mRCC patients who received sunitinib 50 mg/day according to the 4/2 schedule, four soluble biomarkers of sunitinib activity were investigated on days 1 and 28 of each cycle (Deprimo et al., 2007). At the end of the first cycle, plasma concentrations of VEGF and placental growth factor (PlGF) were increased, whereas those of soluble receptors of VEGF (namely sVEGFR2 and sVEGFR3) were decreased with respect to baseline. Of importance, the concentrations of the four soluble biomarkers returned to the baseline values after 2 weeks of rest, suggesting that these effects lasted for a period of time after cessation of drug administration (Deprimo et al., 2007). According to that long-lasting effect of sunitinib, the drug was administered as a neoadjuvant treatment at the dose of 50 mg/day and tumors were excised at various time intervals from the stop of drug intake (Griffioen et al., 2012). Interestingly, biomarkers of angiogenesis, such as circulating endothelial cells and angiopoietin plasma concentrations, rapidly increased after chemotherapy discontinuation, whereas tumor microvessel density remained stable for a long period (approximately 3 weeks) (Griffioen et al., 2012). For sake of completeness, the discontinuation of sunitinib resulted in a significant rebound (“flare”) of cancer cell proliferation detected by positron emission tomography (PET) imaging in a mouse model (Nagengast et al., 2011). A withdrawal flare was also confirmed by PET imaging in 16 patients treated with sunitinib 50 mg/day according to 4/2 or 2/1 schedule (Liu et al., 2011). Those results could support the continuous dosing of sunitinib without rest periods as a further attractive alternative regimen.

It is worth noting that a sigmoidal dose-response curve may describe the relationships between the systemic exposure to the drug (as the area-under-the-curve from time 0 up to the 24th hour, AUC_0−24*h*_), time to progression (TTP) and overall survival (OS) in mRCC patients (Houk et al., 2010). In particular, AUC_0−24*h*_-values higher than 1.5 h × mg/L were related to the highest probability of achieving partial responses (>80%) and disease stabilization (>95%). Those effective concentrations were obtained with sunitinib 50 mg/day in 7–14 days of a 2/1 schedule (Britten et al., 2008), during which the mean AUC-values of the drug increased from 0.341 h × mg/L on day 1 up to 1.717 h × mg/L on day 14. An interesting finding of the study conducted by Britten and colleagues is the presence of measurable sunitinib plasma concentrations at the end of the 7-day rest period (Britten et al., 2008). It is worth noting that doses of 50 mg/day led to a median value of sunitinib plasma AUC of 1.113 h × mg/L, whereas sunitinib 75 mg/day led to median AUC-values of 2.347 h × mg/L (Faivre et al., 2006). Taken into consideration both the large interindividual variability of sunitinib pharmacokinetics (Houk et al., 2009) and the non-linear pharmacokinetics of the drug confirmed in clinical trials (Faivre et al., 2006; Houk et al., 2010), some patients could be exposed to subtherapeutic concentrations, whereas an eventual dose escalation should be cautious. Therefore, regardless the schedule, there is room to dosage optimization thanks to therapeutic drug monitoring protocols.

**Figure 1:**
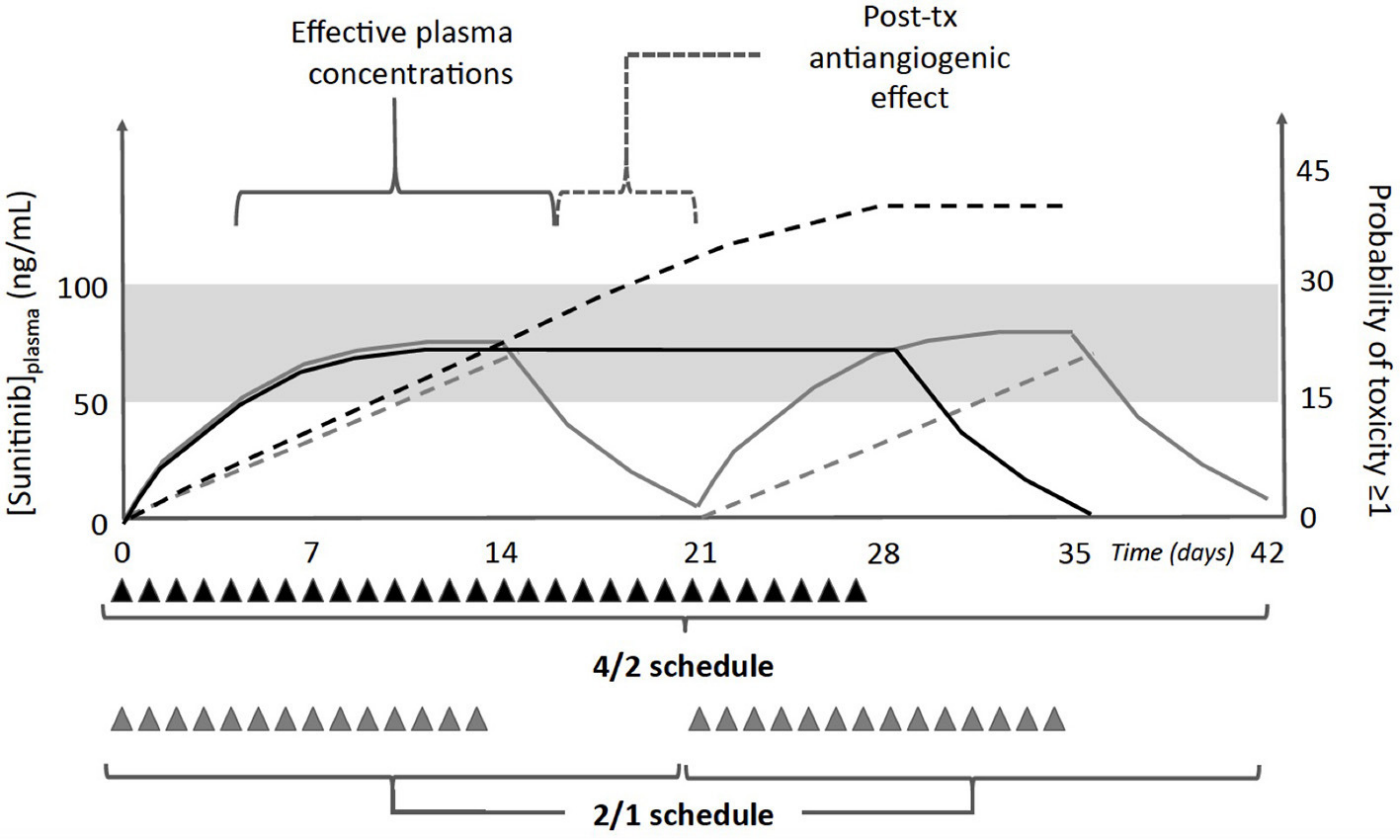
Schematic representation of plasma concentrations (solid lines) and probability of toxicities grade ≥1 (dashed lines) associated with administration of sunitinib 50 mg/day (triangles) according to the standard 4/2 (black lines/triangles), or the alternative 2/1 schedule (gray lines/triangles). Both regimens have the same dose intensity, but the increased severity of toxicities is associated with the standard schedule. Furthermore, there is the evidence that sunitinib effects may extend up to 1 week after the last daily dose (post-tx antiangiogenic effects), hence the 1 week of rest in the 2/1 schedule could be beneficial for patients while ensuring the recovery from mild toxicities. The gray area represents the therapeutic range of sunitinib plasma concentrations (50–100 ng/mL).

On the basis of the available pharmacokinetic and pharmacodynamics data one may argue that the 2/1 schedule is an active treatment because sunitinib achieves effective target plasma concentrations within the range 50–100 ng/mL (Figure 1), and the exposure is maximized to obtain a therapeutic effect (i.e., AUC_0−24_ of approximately 1.5 h × mg/L). Furthermore, the antiproliferative effect of sunitinib still continues during the rest period of 7 days, because (1) the drug is still circulating within the blood flow at the beginning of the next cycle and (2) the final effect on tumor microvessel density does last for days. At this point, the improved tolerability of the 2/1 schedule helps to support its therapeutic advantages with respect to the standard schedule.

## Safety profile

Several studies have compared the tolerability of the standard 4/2 schedule with that of the alternative one. Overall, the conclusion of those studies is the same, with a highly significant reduction of moderate-to-severe toxic effects, namely diarrhea, fatigue, mucositis, thrombocytopenia, and hypertension in the alternative schedule (Bracarda et al., 2015; Gore et al., 2015). In one of those recent papers, patients were shifted from the standard to the alternative schedule and the latter was characterized by a significant improvement in tolerability (Bracarda et al., 2015). As a consequence, a greater percentage of patients continued the treatment without delays or reductions of daily doses, being those events potentially related to a diminished therapeutic effect. Schnadig and colleagues drew the same conclusions (Schnadig et al., 2014), because the occurrence of unmanageable toxicities caused treatment discontinuation or dose reduction, which in turn were associated with inferior clinical outcomes with respect to patients who tolerate sunitinib and remained on treatment.

At this point, the second question is why the 2/1 schedule is better tolerated than the 4/2 one. Both treatments have the same dose intensity in a 6-week period, and both of them have a rest period that allows patients to recover from toxicities. However, during a 4/2 schedule, patients start to experience sunitinib-induced toxicities at the second week of treatment, and the severity usually increases over the next 2 weeks (Najjar et al., 2014). This means that the probability to observe a moderate-to-severe toxicity in the 4/2 treatment is higher in comparison with the alternative schedule 2/1 (Figure 1). In the latter case, sunitinib administration is halted at day 14, before adverse events could worse, and 1 week off treatment is likely enough to allow the complete recovery from mild, low-grade toxic effects. Furthermore, the work of Houk and colleagues helps to explain this hypothesis through a mathematical model (Houk et al., 2010). The rate of incidence and severity of toxicities increases with the length of sunitinib administration, as it occurs for fatigue. In particular, the increase in treatment duration from 14 up to 28 days nearly doubled the probability to experience fatigue ≥1, considering that the calculated half-life for the appearance of this toxicity is 8 days. Therefore, the better tolerability of the 2/1 schedule compared with the 4/2 regimen may be attributable to the lowest probability of occurrence of moderate-to-severe adverse events.

The last point that should be briefly discussed is the duration of the rest period with respect to both treatment tolerability and drug pharmacokinetics. As anticipated above, sunitinib concentrations are measurable at the end of the 7-day rest period (Britten et al., 2008). That result was not surprising because the drug has a long terminal half-life (approximately 70 h), but an accumulation of the drug over the next cycles was firmly excluded (Britten et al., 2008). Therefore, AUC-values associated with a higher therapeutic benefit could be achieved by a 2/1 schedule without increasing the risk of toxic effects caused by the accumulation of the drug over the entire planned treatment.

## Conclusions

The recent studies on the alternative schedule 2/1 have demonstrated an identical efficacy in comparison with the standard 4/2 treatment because both schedules achieve the same plasma levels and share the same dose intensity, but the first one is characterized by a reduced incidence of moderate-to-severe adverse events (Bracarda et al., 2015). The improved tolerability is the major difference between the two schedules. Indeed, mild toxic effects occur with less frequency in the 2/1 regimen, allow a longer treatment duration and, consequently, a longer disease control (Bracarda et al., 2015). On the contrary, any treatment delay or suspension due to adverse reactions after the 4/2 schedule have been associated with a reduced therapeutic benefit and inferior clinical outcomes (Schnadig et al., 2014). Therefore, the better tolerability of the 2/1 schedule increases the possibility of extended treatments, which do represent a therapeutic advantage for mRCC patients. It is worth noting that pharmacological data collected in several preclinical and clinical studies may explain those observations. While offering new areas for discovering predictive biomarkers, pharmacodynamic studies have demonstrated that the antiangiogenic effect of sunitinib follows the short-lived changes of soluble factors and persists after their normalization in circulating blood. The post-chemotherapeutic inhibitory effect on microvessels exerted by sunitinib lasts for a variable period of time after the cessation of drug administration, its duration seems to be long enough to cover the rest period between two consecutive cycles, at least in the 2/1 schedule, despite a withdrawal flare is possible as confirmed by some preclinical and clinical studies. Intriguingly, a pharmacodynamic modeling demonstrated that the increase in toxicity risk doubled nearly every 8 days (Houk et al., 2010), hence explaining why a 2-week treatment is devoid of those severe toxicities reported during the standard schedule. As a consequence, the majority of patients may recover from mild-grade adverse reactions during the rest period, even if it only lasts a week.

In conclusion, it is really impressive how changing the schedule of drug administration by splitting the treatment into two periods has increased tolerability beyond expectancies, hence demonstrating the possibility to optimize tolerability and subsequently efficacy by changing the standard regimens in a rational way.

## Author contributions

ADP, SB, EA, and RD contributed to the analysis of pertinent bibliography and critically revised the manuscript. ADP is accountable for all aspects of the work to ensure accuracy and truthfulness. All of the authors approved the manuscript.

### Conflict of interest statement

ADP served as a board member for Novartis Farma SpA. The other authors declare that the research was conducted in the absence of any commercial or financial relationships that could be construed as a potential conflict of interest.
